# ConFIRM trial - conversion of in vitro fertilization cycles to intrauterine inseminations in patients with a poor ovarian response to stimulation: a protocol for a multicentric, prospective randomized trial

**DOI:** 10.1186/s13063-018-2936-5

**Published:** 2018-10-17

**Authors:** Léa Delbos, Elsa Parot-Schinkel, Hady El Hachem, Guillaume Legendre, Philippe Descamps, Lisa Boucret, Véronique Ferré-L’Hotellier, Pauline Jeanneteau, Cécile Dreux, Catherine Morinière, Pascale May-Panloup, Pierre-Emmanuel Bouet

**Affiliations:** 1 Department of Reproductive Medicine, Angers University Hospital, 4 rue Larrey, 49000 Angers, France; 2 Clinical Research Unit, Angers University Hospital, Angers, France; 3 Department of Reproductive Medicine, Clemenceau Medical Center, Beirut, Lebanon; 4 Department of Reproductive Medicine, Pointe-à-Pitre University Hospital, Guadeloupe, France

**Keywords:** IVF, Conversion to IUI, Poor ovarian response

## Abstract

**Background:**

To date, there is no consensus on the ideal management strategy of patients with poor ovarian response (POR) to controlled ovarian stimulation (COS) for in vitro fertilization (IVF). Currently, these patients are given the choice of: (1) canceling the cycle; (2) proceeding with COS regardless of the poor response, and performing the oocyte retrieval and transfer of embryos when available; or (3) conversion to an intrauterine insemination (IUI). When the decision to proceed with the COS cycle is taken, it is not clear whether IVF or conversion to IUI is the best choice. If live birth rates were comparable between the two strategies, conversion to IUI would be the better option for poor responders, since it is less invasive and is associated with a lower cost.

**Methods:**

We designed a non-inferiority, multicentric, randomized controlled trial that will be conducted in 18 French Reproductive Medicine centers. We defined POR as the presence of only two or four mature follicles ≥ 14 mm on ovulation trigger day. Patients with POR will be randomized into two parallel arms: “IVF” and “conversion to IUI.” Our main objective is to compare the efficiency of IVF and conversion to IUI in patients with POR to COS. The primary outcome is the live birth rate, defined as the birth of a living infant after 22 weeks’ gestational age, or weighing ≥ 500 g. One of the secondary objectives is to compare the cost-efficiency of both strategies at 12 months. We will need to include 940 patients (470 in each arm), and the duration of the inclusion period is estimated to be 36 months.

**Discussion:**

This is the first randomized controlled trial to compare the outcomes of IVF and embryo transfer to conversion to IUI in patients with POR to COS. If our study shows that conversion to IUI is non-inferior to IVF in terms of clinical efficiency and live birth rate, it would confirm IUI as a better alternative for patients, both individually (less invasive and more patient-friendly) and collectively (lower cost).

**Trials registration:**

ClinicalTrials.gov, ID: NCT03362489. Registered on January 10th, 2018.

**Electronic supplementary material:**

The online version of this article (10.1186/s13063-018-2936-5) contains supplementary material, which is available to authorized users.

## Background

In vitro fertilization (IVF), with or without intracytoplasmic spermatozoid injection (ICSI), followed by embryo transfer, is commonly offered for millions of couples with infertility worldwide. It is usually preceded by controlled ovarian stimulation (COS) with exogenous gonadotropins, which allows to increase the number of follicles and mature oocytes available, thus increasing the number of good-quality embryos available for transfer, and improving the pregnancy and live birth rates (LBRs). However, in about 6% of COS cycles for IVF, less than five mature follicles are obtained despite the use of high doses of exogenous gonadotropins [1, 2]. These cases are referred to as “poor ovarian response to stimulation” (POR), and are associated with lower pregnancy and LBRs. When managing patients with POR during a COS cycle, there are three available options. The first is to proceed with the stimulation despite the poor response, and perform the oocyte retrieval and transfer the available embryos. However, there is a high risk of failure to retrieve oocytes and the absence of embryos to transfer, which should be weighed against the risks of complications associated with the oocyte retrieval and anesthesia. Moreover, such events could be the source of significant disappointment and stress for the couple, and they should be thoroughly counseled before proceeding with the stimulation [3, 4]. The second option is conversion of the IVF cycle to an intrauterine insemination (IUI) in patients having at least one patent tube and adequate semen parameters [5, 6]. Conversion to IUI allows the couple to benefit from the treatments already administered, without the risk of an oocyte retrieval/embryo transfer failure. Finally, the third option is to cancel the IVF cycle. The decision to cancel a cycle is always a challenging one, since it involves not only clinical, but also emotional and financial considerations. Indeed, it is often difficult to announce to a couple the decision to abandon a COS cycle following several weeks of preparation, and after the patient has had several days of high-dose gonadotropin injections. Moreover, several studies have shown that in women with POR, it remains advantageous to proceed with the planned assisted reproductive treatment (ART) [7].

In 2011, the European Society of Human Reproduction and Embryology (ESHRE) published the Bologna criteria in order to “standardize the definition of POR in a simple and reproducible manner” [3, 4]. At least two of the following three criteria are required to establish the definition of POR: (1) advanced maternal age (> 40 years) or any other risk factor for POR; (2) a previous POR (at least three oocytes with a conventional stimulation protocol); (3) an abnormal ovarian reserve test (i.e., antral follicle count (AFC) less than five to seven follicles or anti-Müllerian hormone (AMH) below 0.5–1.1 ng/mL). Moreover, two episodes of POR after maximal stimulation are enough to define a patient as a poor responder according to the Bologna criteria, in the absence of advanced maternal age or abnormal ovarian reserve [3]. However, ever since their publication, these criteria have been the subject of many debates and several revisions have been suggested and requested [5]. Indeed, some aspects have been criticized, such as the supposed homogeneity of the population, the defined thresholds for age, AFC and AMH, the proposed risk factors other than age, and the lack of consideration for oocyte quality compared to quantity [5].

Only five studies compared the outcome of IVF and conversion to IUI in patients with POR [2, 8–11]. These studies have reported that there is no benefit in proceeding with IVF in the presence of only one mature follicle. However, the results in cases with two, three or four mature follicles were divergent. Indeed, three studies reported the superiority of IVF [2, 8, 9], whereas the two others failed to show a difference between IVF and conversion to IUI [10, 11]. Unfortunately, these studies have low levels of evidence, mostly because of their retrospective observational designs, and the presence of multiple biases [2, 8–11]. Therefore, the ideal management of patients with two, three or four mature follicles following COS remains unknown [12]. This is why we have decided to perform the first randomized controlled trial (RCT) to compare these two treatment methods. If our study shows that conversion to IUI is non-inferior to IVF in terms of clinical efficiency and LBR, it would confirm IUI as a better alternative for patients, both individually (less invasive and more patient-friendly) and collectively (lower cost).

## Methods

### Aims and outcome measures

The primary objective is to compare the efficiency of IVF and conversion to IUI in patients with a poor ovarian response to COS. The main outcome measure is the LBR, defined as the birth of a living infant after 22 weeks’ gestational age (GA), or weighing ≥ 500 g.

The secondary objectives are: (1) to compare the outcomes of IVF and conversion to IUI in women with POR to COS: biochemical pregnancy rate, clinical pregnancy rate, pregnancy loss rate, multiple pregnancy rate, mean term at delivery and postnatal outcomes; (2) to compare the impacts of IVF and conversion to IUI on overall outcomes in women with POR to COS, according to the number of mature follicles on trigger day (2 vs 3 vs 4), and according to age (< 40 years vs ≥40 years); (3) to compare the impacts of IVF and conversion to IUI on the cumulative clinical pregnancy and LBRs – taking into account frozen embryo transfers in IVF – in women with POR to COS; (4) to compare the clinical efficiency of IVF and conversion to IUI in women considered “poor ovarian responders” according to the Bologna criteria; (5) to analyze the rate of IVF cycles with failed oocyte retrievals (no oocytes) and no embryo transfers and (6) to compare the cost-efficiency of both strategies at 12 months.

The secondary outcomes measures are:

- Biochemical pregnancy rate: defined as serum human chorionic gonadotropin (HCG) levels > 10 IU/L, 14 days after the IUI or the embryo transfer, followed by a rapid decrease until being undetectable
- Clinical pregnancy rate: defined as fetal cardiac activity at 6–7 weeks’ GA
- Spontaneous pregnancy loss (PL) rate: including early and late pregnancy losses
- Multiple pregnancy rate: defined as more than two embryos visualized on ultrasound at 7 weeks’ GA
- Term at delivery, neonatal complications and survival
- All outcome measures will be further analyzed according to patients’ age (< 40 years vs ≥40 years) and the number of follicles on trigger day (2 vs 3 vs 4)
- All outcome measures will be further analyzed in the subgroup of women considered poor responders according to the Bologna criteria
- The rate of IVF cycles with failed oocyte retrievals and no embryo transfers
- Cumulative clinical pregnancy and LBRs in the IVF group, taking into account fresh and frozen embryos transferred in subsequent cycles
- Cost-efficiency analysis at 12 months

### Trial design

This is a prospective, multicenter, parallel-group, open-label randomized controlled and non-inferiority trial conducted at 18 French departments of reproductive medicine (Table 1).

**Table 1:** French partners of study

Gynecologists	Center/department
Dr Pierre-Emmanuel Bouet	Coordinating investigator, Angers University Hospital, Angers
Dr Florence Leperlier	Nantes University Hospital, Nantes
Dr Claire de Vienne	Caen University Hospital, Caen
Dr Nathalie Massin	Créteil University Hospital, Créteil
Dr Mathilde Domin-Bernhard	Rennes University Hospital, Rennes
Dr Marion Cornuau	Tours University Hospital, Tours
Dr Olivier Pirrello	Strasbourg University Hospital, Strasbourg
Dr Catherine Morinière	Pointe-à-Pitre University Hospital, Guadeloupe
Dr Sophie Fressard	Lorient Public Hospital, Lorient
Dr Marie-Laure Langlois	Clinique Jules Verne, Nantes
Dr Anne-Cécile Racine	Polyclinique de l’Atlantique, Saint Herblain
Dr Anne Guivarc’h-Levêque	Clinique de la Sagesse, Rennes
Dr Frédéric Lamazou	Clinique Pierre Cherrest, Neuilly-sur-Seine
Dr Xenia Lechat	Polyclinique Jean Vilar, Bruges
Dr Claudine Vasseur	Clinique Léonard de Vinci, Chambray-lès-Tours
Dr Aurore Guennifey	Grenoble University Hospital, Grenoble
Pr Blandine Courbière	Marseille University Hospital, Marseille
Pr Michaël Grynberg	Antoine Béclère Hospital, Clamart

The study protocol was designed using the recommendations of the Consolidated Standards of Reporting Trial (CONSORT) Statement, Recommendations for Interventional Trials (SPIRIT) 2013 Checklist (Additional file 1) and according to the guidelines of cost-effectiveness studies of the French Health Authority. Eligible patients will be randomized equally to either conversion to IUI or IVF.

### Recruitment

**Fig. 1:**
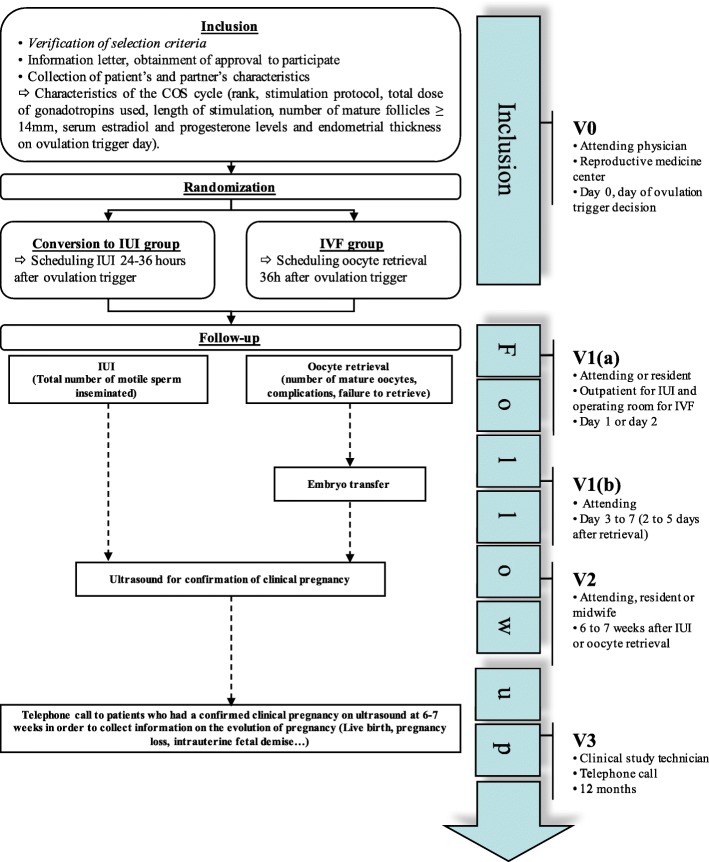
Flow chart. *COS* controlled ovarian stimulation, *V* visit, *IUI* intrauterine insemination, *IVF* in vitro fertilization

Patients will be recruited in all participating reproductive medicine centers across France. Potentially eligible patients will be pre-identified by the investigator or the co-investigators, based on their medical files and the different selection criteria that can be evaluated at the time, without informing these patients. Patients will be identified during ultrasound monitoring of follicular growth in the course of COS, and those with POR will be handed an information letter explaining the study protocol, so that they are already fully informed by the time they reach ovulation trigger day. The inclusion visit will take place on the day the decision to trigger ovulation is taken. During the inclusion visit, the investigator will thoroughly explain the study and hand the patient an information letter, written in an easily accessible language. The patient who wishes to be enrolled will sign the consent form, and will be randomized to one of the two groups. Randomization will be performed centrally by the trial coordinators using an online randomization module (Ennov Clinical®) in a 1:1 allocation ratio between IVF and conversion to IUI, as shown in Fig. 1. Moreover, randomization will be dynamic, and stratified by: center, number of mature follicles (two, three or four) and age (< 40 or ≥ 40 years).

### Eligibility criteria

The inclusion criteria are: (1) patients who accepted being included and signed the consent forms; (2) age ≥ 18 and < 43 years and (3) IVF cycle, with and without ICSI, using the “conventional” agonist (long and short) or antagonist protocol, using urinary or recombinant gonadotropins, and having only two, three or four mature follicles (≥ 14 mm) on the ovulation trigger day.

The exclusion criteria are: (1) confirmed bilateral tubal occlusion; (2) non-French speaking patients; (3) partners with severe oligoasthenoteratospermia (OATS) (less than five million motile spermatozoa in the ejaculate); (4) suboptimal stimulation protocols: protocols ≤ 150 IU of daily gonadotropins or mild stimulation protocols or natural and modified natural cycle protocols; (5) couples undergoing IVF for Preimplantation Genetic Diagnosis (PGS) or Preimplantation Genetic Screening (PGS); (6) women under legal guardianship; (7) women with no health or social security coverage and (8) women participating in other interventional trials.

### Study population

At our center, in 2014, we performed 30 IVF cycles following COS with high doses of gonadotropins (≥ 300 IU) in patients at risk of POR. Our clinical pregnancy rate (CPR) was 10% (3/30), and our LBR was 10% (3/30). During that same year, we had 37 IVF cycles which were converted to IUI because of POR. The CPR was 11% (4/37) and LBR was 5% (2/37). If we consider that a maximal difference of 5% between IVF and conversion to IUI is clinically acceptable in order to consider conversion to IUI to be non-inferior to IVF, we will need to include 940 cycles, 470 in each group, to confirm the non-inferiority, with a power of 80%, an α risk of 5% and with 5% of non-evaluable cycles. It is worth noting that patients who are excluded from the study for failure of treatment can be included later on during their next IVF cycle, considering they still fulfill the inclusion criteria.

### Procedures

Patients will be recruited on the day of ovulation trigger. Ovulation will be triggered with an injection of urinary HCG (administered intramuscularly or subcutaneously, 5000 or 10,000 units), or recombinant HCG, depending on the participating center’s protocol.

**Fig. 2:**
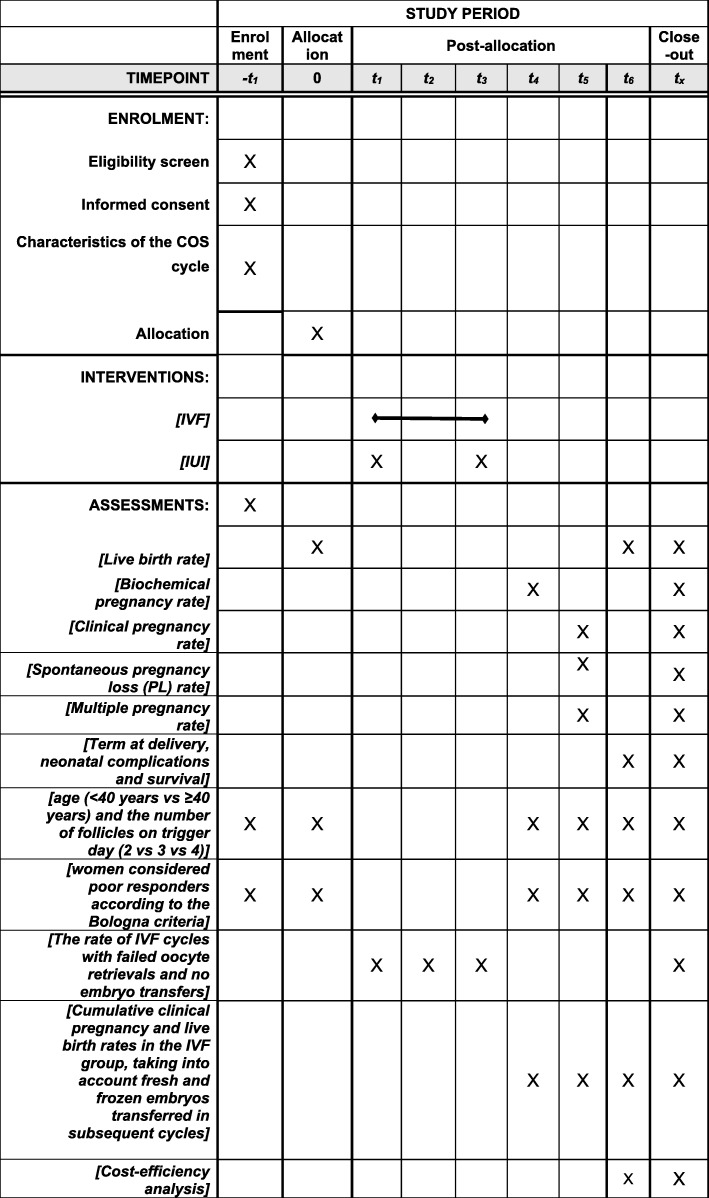
Standard Protocol Items: Recommendations for Interventional Trials (SPIRIT) Figure of ConFIRM study protocol
- In the “conversion to IUI” arm (intervention group), IUI is performed 24 to 36 h after ovulation trigger. The partner provides the sperm on site and on the morning of the insemination, and it will be prepared with a two-layer density gradient. Attending physicians, fellows or residents will perform the insemination in the outpatient department, using a soft catheter, with the patient lying in the gynecologic position

Patients will be randomized into two parallel arms (Fig. 2):

- In the “IVF” arm, oocyte retrieval is performed 36 h after the HCG injection, in the operating room, under transvaginal ultrasound guidance, under local or general anesthesia. The procedure lasts about 20 min and the patients are discharged on the same day. The oocytes retrieved from the follicles are transported immediately to the laboratory for fertilization with the partner’s sperm. Fertilization is done either via conventional IVF, or via ICSI, depending on the indication. Embryos are later transferred into the uterus on day 3 or day 5, under ultrasound guidance, in the outpatient department

### Cost-effectiveness analysis

One of our secondary objective is to perform a cost-effectiveness analysis, in order to determine the optimal treatment strategy for patients with POR to COS, not only in terms of efficiency, but also in terms of cost. We will compare the reference strategy (IVF) to the experimental strategy (conversion to IUI). We will use the incremental cost-effectiveness ratio (ICER), which represents the average incremental cost associated with one additional unit of the measure of effect. IECR is calculated as follows: IECR = *Δ* cost / *Δ* effectiveness. Costs will include the pharmacological compounds, the IVF and IUI procedures, the hospitalization and the potential complications, and will be expressed in euros (€). Moreover, the loss of productivity sustained by the women and their partners during the treatment will also be included in the costs. We will not include the hospital resources used for the COS monitoring (ultrasound scans, blood tests…), nor the ones for the pregnancy follow-up since the treatment method has no impact on the evolution of pregnancy once a clinical pregnancy is confirmed. We will perform a univariate sensitivity analysis (Tornado diagram) to assess the effects of several parameters on the overall outcome.

### Data collection and follow-up procedures

All data will be recorded in an electronic Case Report Form (eCRF) specifically elaborated for the study (eCRF CleanWEB, Telemedicine Technologies S.A.S) and will be collected at four specific moments: upon randomization (V0), when performing the procedure (IVF or IUI) (V1), after the ultrasound at 6–7 weeks’ GA to confirm the clinical pregnancy (V2) and after a telephone call 12 months later for follow-up of women who had a clinical pregnancy confirmed at 6–7 weeks (V3). In the IVF group, the procedure (V1) will be divided into: V1a: day of oocyte retrieval, and V1b: day of embryo transfer. In the conversion to IUI group, data collection will be on the day of insemination (Fig. 1).

### Statistical analysis

We will perform a descriptive analysis of the population’s characteristics. Categorical variables will be expressed as numbers with percentages, and compared using Pearson’s chi-square test or Fisher’s exact test. Continuous variables will be reported as mean values and standard deviations, or medians with 25th and 75th percentile, and compared using Student’s *t* test or Mann-Whitney’s non-parametric test. All statistical tests will be bilateral and a *p* value < 0.05 will be considered statistically significant. We will perform an intention-to-treat and a per-protocol analysis of all outcomes. For the primary outcome, conversion to IUI will be considered non-inferior to IVF if the upper bound of the one-sided 95% confidence interval of the difference in LBRs between the two arms (the rate in IVF minus the rate in conversion to IUI) is ≤ 5%. Analysis of the primary and secondary outcomes will be adjusted according to the stratification variables used for randomization. The primary outcome will be tested using the raw difference in LBRs, and no complex model incorporating the stratification variables will be used. The dynamic randomization on these variables will ensure the variables are well-balanced. Subgroup analysis will be performed according to patients’ age (< 40 vs ≥40 years), the number of mature follicles (two vs three vs four) and patients considered poor ovarian responders according to the Bologna criteria. On the other hand, the cost-effectiveness analysis will be performed on an intention-to-treat basis (the hypothesis being that conversion to IUI is superior to IVF).

### Data circulation

All data from the trial will be compiled in an eCRF. Only people involved in the trial will have access to the data, via a username and a password. No patient identifying information will be stored, and only the first letter of the surname and of the last name will be kept, without any mention of the full name or date of birth. Patient code will be composed of the participating center’s number and another number assigned by that center. Each participating center will store its own data for the duration set by regulation for this type of study under the center’s investigator’s responsibility. Only investigators from Angers University Hospital (AUH) will have access to patient data from other centers on the eCRF. Therefore, for centers that cannot complete patient follow-up at 12 months, patients’ coordinates will be retrieved from the eCRF by AUH investigators and personnel in charge of patient follow-up. At the end of the trial, all data will be deleted. Approval from the CCTIRS (Comité consultatif sur le traitement de l’information en matière de recherche) (Advisory Committee on Treatment of Information in Research in the Field of Healthcare) and the CNIL (Commission nationale informatique et liberté) (National Commission on Informatics and Liberty) have been requested.

### Monitoring and adverse events

We do not expect to have undesirable or serious undesirable events related to the study and its proceedings. Indeed, all procedures included in this study (COS and its monitoring, ovulation trigger, oocyte retrieval, embryo transfer, IUI) are standard procedures and will be performed in accordance with the national guidelines and recommendations. The only part that is specific to our RCT, and that is not considered part of routine practice, is the telephone call that will be made to patients 12 months after enrollment in order to collect information on the evolution of pregnancy. This might be a sensitive topic, especially for couples who did not have a healthy live birth because of pregnancy loss or complications of pregnancy. The personnel in charge of this part will be trained in how to approach the couples and how to deal with the special situations. Moreover, we will have a psychologist available for couples to consult when needed.

All adverse events related to the oocyte retrieval (bleeding, hemoperitoneum, infection, peritonitis, pelvic abscess, etc.) and the anesthesia during the retrieval will be recorded. We will also document whether the complications required any additional treatment, procedure or hospitalization, and add them to the treatment cost.

### Expected repercussions

If LBRs were to be comparable between IVF and conversion to IUI in patients with POR to COS, and if our cost-effectiveness analysis confirms that IUI is indeed more cost-effective, then we would confirm that conversion to IUI is the better treatment option, since it is also less invasive.

## Discussion

This is the first RCT to compare the outcomes of IVF and embryo transfer to conversion to IUI in patients with poor ovarian response (POR) to controlled ovarian stimulation (COS). If our study shows that conversion to IUI is non-inferior to IVF in terms of clinical efficiency and LBR, it would confirm IUI as a better alternative for patients, both individually (less invasive and more patient-friendly) and collectively (lower cost).

## Trial status

The trial already began on 10 January 2018, and participant recruitment and follow-up will continue over a 48-month period, with the anticipated final follow-up telephone call(s) occurring in January 2022. Primary analyses will be complete by June 2022.

## Additional file

Additional file 1: Standard Protocol Items: Recommendations for Interventional Trials (SPIRIT) 2013 Checklist: recommended items to address in a clinical trial protocol and related documents. (DOCX 74 kb)

## Abbreviations

AFC: Antral follicle count
AMH: Anti-Müllerian hormone
ART: Assisted reproductive treatment
AUH: Angers University Hospital
CCTIRS: Comité consultatif sur le traitement de l’information en matière de recherché = Advisory Committee on Treatment of Information in Research in the Field of Healthcare
CNIL: Commission nationale informatique et liberté = National Commission on Informatics and Liberty
CONSORT: Consolidated Standards of Reporting Trial
COS: Controlled ovarian stimulation
CPR: Clinical pregnancy rate
eCRF: Electronic Case Report Form
ESHRE: European Society of Human Reproduction and Embryology
GA: Gestational age
HCG: Human chorionic gonadotropin
ICER: Incremental cost-effectiveness ratio
ICSI: Intracytoplasmic spermatozoid injection
IUI: Intrauterine insemination
IVF: In vitro fertilization
LBR: Live birth rate
OATS: Oligoasthenoteratospermia
PL: Pregnancy loss
POR: Poor ovarian response
RCT: Randomized controlled trial

## References

[1] Abusheikha N, Lass A, Burnley A (2001). In vitro fertilization cycles converted to intrauterine insemination because of poor follicular response have low success rates. Fertil Steril.

[2] Reichman DE, Gunnala V, Meyer L (2013). In vitro fertilization versus conversion to intrauterine insemination in the setting of three or fewer follicles: how should patients proceed when follicular response falls short of expectation?. Fertil Steril.

[3] Ferraretti AP, Marca AL, Fauser BCJM (2011). ESHRE consensus on the definition of “poor response” to ovarian stimulation for in vitro fertilization: the Bologna criteria. Hum Reprod.

[4] Younis JS, Ben-Ami M, Ben-Shlomo I (2015). The Bologna criteria for poor ovarian response: a contemporary critical appraisal. J Ovarian Res.

[5] Badawy A, Wageah A, El Gharib M (2011). Prediction and diagnosis of poor ovarian response: the dilemma. J Reprod Infertil.

[6] Ardenti R, Campari C, Agazzi L (1999). Anxiety and perceptive functioning of infertile women during in-vitro fertilization: exploratory survey of an Italian sample. Hum Reprod Oxf Engl.

[7] Norian JM, Levens ED, Richter KS (2010). Conversion from assisted reproductive technology to intrauterine insemination in low responders: Is it advantageous?. Fertil Steril.

[8] Quinquin M, Mialon O, Isnard V (2014). In vitro fertilization versus conversion to intrauterine insemination in Bologna-criteria poor responders: how to decide which option?. Fertil Steril.

[9] Nicopoullos JDM, Abdalla H (2011). Poor response cycles: when should we cancel? Comparison of outcome between egg collection, intrauterine insemination conversion, and follow-up cycles after abandonment. Fertil Steril.

[10] Shahine LK, Lathi RB, Baker VL (2009). Oocyte retrieval versus conversion to intrauterine insemination in patients with poor response to gonadotropin therapy. Fertil Steril.

[11] Freour T, Dubourdieu S, Mirallie S (2010). IVF conversion to IUI in poor responders: an observational study. Arch Gynecol Obstet.

[12] Bouet PE, Legendre G, Delbos L (2018). IVF versus conversion to IUI in patients with poor ovarian response to controlled ovarian stimulation. Gynecol Obstet Fertil Senol.

