# Supplementary material for: ConFIRM trial - conversion of in vitro fertilization cycles to intrauterine inseminations in patients with a poor ovarian response to stimulation: a protocol for a multicentric, prospective randomized trial
Source: Trials. 2018 Oct 17;19:565. doi: 10.1186/s13063-018-2936-5 (PMC6192099; doi:10.1186/s13063-018-2936-5)
Supplement: Supplementary file 1 — Standard Protocol Items: Recommendations for Interventional Trials (SPIRIT) 2013 Checklist: recommended items to address in a clinical trial protocol and related documents. (DOCX 74 kb) [file 13063_2018_2936_MOESM1_ESM.docx]

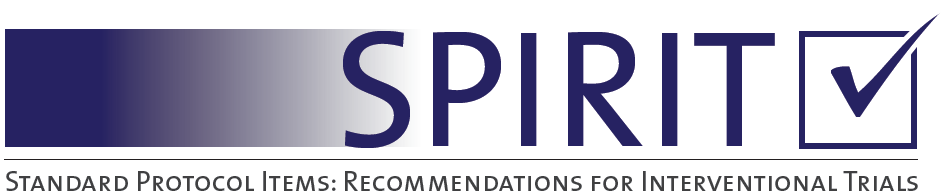


SPIRIT 2013 Checklist: Recommended items to address in a clinical trial protocol and related documents*

| Section/item | ItemNo | ClinicalTrials.gov : TRLS-D-18-00386R1 | |
| --- | --- | --- | --- |
| **Administrative information** | | | |
| Title | 1 | ConFIRM trial: conversion of in vitro fertilization cycles to intrauterine inseminations in patients with a poor ovarian response to stimulation. A protocol for a multicentric prospective randomized trial. | |
| Trial registration | 2a | ClinicalTrials.gov: Conversion of in Vitro Fertilization Cycles to Intrauterine Inseminations in Patients With a Poor Ovarian Response to Stimulation (ConFIRM). NCT03362489, December 5th, 2017  *Trial identifier and registry name. If not yet registered, name of intended registry* | |
|  | 2b | N/A  *All items from the World Health Organization Trial Registration Data Set* | |
| Protocol version | 3 | Version 5: 25/01/2018  *Date and version identifier* | |
| Funding | 4 | The research project is funded by le Programme Hospitalier de Recherche Clinique Interrégional du Grand Ouest de 2016 n° 49RC17_0027/ LOI API16/A/042.  *Sources and types of financial, material, and other support* | |
| Roles and responsibilities | 5a | Pierre Emmanuel Bouet^1^, Elsa Parot-Schinkel^2^, Léa Delbos1, Catherine Morinière^4^ and Pascale May-Panloup^1^ conceived and designed, included the power calculation. Pierre Emmanuel Bouet and Elsa Parot-Schinkel acquired legal authorizations. The study protocol and manuscript have been drafted by Hady El Hachem^3^, Guillaume Legendre^1^, Philippe Descamps^1^, Lisa Boucret^1^, Véronique Ferré-L’Hotellier^1^, Pauline Jeanneteau^1^, Cécile Dreux^1^. All authors read and approve the final manuscript.  ^1^Department of Reproductive Medicine, Angers University Hospital, Angers, France.  ^2^Clinical Research Unit, Angers University Hospital, Angers, France.  ^3^Department of Reproductive Medicine, Clemenceau Medical Center, Beirut, Lebanon.  ^4^Department of Reproductive Medicine, Pointe-à-Pitre University Hospital, Guadeloupe, France.  *Names, affiliations, and roles of protocol contributors* | |
|  | 5b | No trial sponsor | |
|  | 5c | No role of study sponsor and funders in study design; collection, management, analysis, and interpretation of data; writing of the report; and the decision to submit the report for publication, including whether they will have ultimate authority over any of these activities | |
|  | 5d | Methodologist: Elsa Parot  Cost-efficiency analysis: Astrid Darsonval  Biostatistician: Bruno Vielle  Data manager: JM Chretien | |
| Introduction |  |  | |
| Background and rationale | 6a | To date, there is no consensus on the ideal management strategy of patients with poor ovarian response (POR) to controlled ovarian stimulation (COS) for *in vitro* fertilization (IVF). Currently, these patients are given the choice of: 1) canceling the cycle; 2) proceeding with COS regardless of the poor response, and performing the oocyte retrieval and transfer of embryos when available; 3) Conversion to an intrauterine insemination (IUI). When the decision to proceed with the COS cycle is taken, it is not clear whether IVF or conversion to IUI is the best choice. | |
|  | 6b | *CF 6a* | |
| Objectives | 7 | | Therefore, the ideal management of patients with 2, 3 or 4 mature follicles following COS remains unknown.[12] This is why we have decided to perform the first RCT to compare these two treatment methods. If our study shows that conversion to IUI is non-inferior to IVF in terms of clinical efficiency and live birth rate, it would confirm IUI as a better alternative for patients, both individually (less invasive and more patient-friendly) and collectively (lower cost). |
| Trial design | 8 | We designed a non-inferiority, multicentric, randomized controlled trial. Randomization will be performed centrally by the trial coordinators using an online randomization module (Ennov Clinical®) in a 1:1 allocation ratio between IVF and conversion to IUI, as shown in Fig. 1. Moreover, randomization will be dynamic, and stratified by: center, number of mature follicles (2, 3 or 4), and age (< 40 or ≥ 40 years). | |
| Methods: Participants, interventions, and outcomes | | | |
| Study setting | 9 | The study will be conducted in 18 French Reproductive Medicine centers. With Centre Hospitalier Universitaire de Angers as referral center. | |
| Eligibility criteria | 10 | The inclusion criteria are: (1) patients who accepted being included and signed the consent forms; (2) age ≥18 and <43 years; (3) IVF cycle, with and without ICSI, using the “conventional” agonist (long and short) or antagonist protocol, using urinary or recombinant gonadotropins, and having only 2, 3 or 4 mature follicles (≥14 mm) on ovulation trigger day.  The exclusion criteria are: (1) confirmed bilateral tubal occlusion; (2) non-French speaking patients; (3) partners with severe oligoasthenoteratospermia (OATS) (<5 millions motile spermatozoa in the ejaculate); (4) suboptimal stimulation protocols : protocols ≤ 150 IU of daily gonadotropins or mild stimulation protocols or natural and modified natural cycle protocols; (5) Couples undergoing IVF for Preimplantation Genetic Diagnosis (PGS) or Preimplantation Genetic Screening (PGS) ; (6) women under legal guardianship; (7) women with no health or social security coverage; (8) women participating in other interventional trials. | |
| Interventions | 11a | Patients will be recruited on the day of ovulation trigger. Ovulation will be triggered with an injection of urinary HCG (intramuscular or subcutaneous, 5 000 or 10 000 units), or recombinant HCG, depending on the participating center’s protocol.  Patients will be randomized into two parallel arms:   - In the “IVF” arm, oocyte retrieval is performed 36 hours after the HCG injection, in the operating room, under transvaginal ultrasound guidance, under local or general anesthesia. The procedure lasts about 20 minutes and the patients are discharged on the same day. The oocytes retrieved from the follicles are transported immediately to the lab for fertilization with the partner’s sperm. Fertilization is done either via conventional IVF, or via ICSI, depending on the indication. Embryos are later transferred into the uterus on day 3 or day 5, under ultrasound guidance, in the outpatient department. - In the “conversion to IUI” arm (intervention group), IUI is performed 24 to 36 hours after ovulation trigger. The partner provides the sperm on site and on the morning of the insemination, and it will be prepared with a two-layer density gradient. Attending physicians, fellows or residents will perform the insemination in the outpatient department, using a soft catheter, with the patient lying in the gynecologic position. | |
|  | 11b | Not applicable | |
|  | 11c | Not applicable | |
|  | 11d | Not applicable | |
| Outcomes | 12 | The primary objective is to compare the efficiency of IVF and conversion to IUI in patients with a poor ovarian response to controlled ovarian stimulation. The main outcome measure is the live birth rate, defined as the birth of a living infant after 22 weeks gestational age (GA), or weighing ≥ 500 g.  The secondary objectives are: (1) to compare the outcomes of IVF and conversion to IUI in women with POR to COS: biochemical pregnancy rate, clinical pregnancy rate, pregnancy loss rate, multiple pregnancy rate, mean term at delivery and postnatal outcomes; (2) to compare the impacts of IVF and conversion to IUI on overall outcomes in women with POR to COS, according to the number of mature follicles on trigger day (2 vs 3 vs 4), and according to age (<40 years vs ≥40 years) ; (3) to compare the impacts of IVF and conversion to IUI on the cumulative clinical pregnancy and live birth rates – taking into account frozen embryo transfers in IVF – in women with POR to COS; (4) to compare the clinical efficiency of IVF and conversion to IUI in women considered “poor ovarian responders” according to the Bologna criteria; (5) to analyze the rate of IVF cycles with failed oocyte retrievals (no oocytes) and no embryo transfers; (6) to compare the cost-efficiency of both strategies at 12 months.  The secondary outcomes measures are:   - Biochemical pregnancy rate: defined as serum HCG levels >10 IU/L, 14 days after the IUI or the embryo transfer, followed by a rapid decrease until being undetectable. - Clinical pregnancy rate: defined as fetal cardiac activity at 6-7 weeks GA. - Spontaneous pregnancy loss (PL) rate: including early and late pregnancy losses. - Multiple pregnancy rate: defined as more than two embryos visualized on ultrasound at 7 weeks GA. - Term at delivery, neonatal complications and survival. - All outcome measures will be further analyzed according to patients’ age (<40 years vs ≥40 years) and the number of follicles on trigger day (2 vs 3 vs 4). - All outcome measures will be further analyzed in the subgroup of women considered poor responders according to the Bologna criteria. - The rate of IVF cycles with failed oocyte retrievals and no embryo transfers. - Cumulative clinical pregnancy and live birth rates in the IVF group, taking into account fresh and frozen embryos transferred in subsequent cycles. - Cost-efficiency analysis at 12 months. | |
| Participant timeline | 13 | See Figure 1 and 2 with Spirit-Figure. | |
| Sample size | 14 | If we consider that a maximal difference of 5% between IVF and conversion to IUI is clinically acceptable in order to consider conversion to IUI to be non-inferior to IVF, we will need to include 940 cycles, 470 in each group, to confirm the non-inferiority, with a power of 80%, an α risk of 5%, and with 5% of non-evaluable cycles. It is worth noting that patients who are excluded from the study for failure of treatment can be included later on during their next IVF cycle, considering they still fulfill the inclusion criteria.  The trial already began on January 10th, 2018, and participant recruitment and follow-up will continue over a 48-months period, with the anticipated final follow-up phone call(s) occurring in January 2022. Primary analyses will be complete by June 2022. | |
|  |  |  | |
| Recruitment | 15 | Patients will be recruited in all participating reproductive medicine centers across France. Potentially eligible patients will be pre-identified by the investigator or the co-investigators, based on their medical files and the different selection criteria that can be evaluated at the time, without informing these patients. Patients will be identified during ultrasound monitoring of follicular growth in the course of COS, and those with POR will be handed an information letter explaining the study protocol, so that they are already fully informed by the time they reach ovulation trigger day. The inclusion visit will take place on the day the decision to trigger ovulation is taken. During the inclusion visit, the investigator will thoroughly explain the study and hand the patient an information letter, written in an easily accessible language. The patient who wishes to be enrolled will sign the consent form, and will be randomized to one of the two groups. Randomization will be performed centrally by the trial coordinators using an online randomization module (Ennov Clinical®) in a 1:1 allocation ratio between IVF and conversion to IUI, as shown in Fig. 1. Moreover, randomization will be dynamic, and stratified by: center, number of mature follicles (2, 3 or 4), and age (< 40 or ≥ 40 years). | |
| **Methods: Assignment of interventions (for controlled trials)** | | | |
| Allocation: |  |  | |
| Sequence generation | 16a | The patient who wishes to be enrolled will sign the consent form, and will be randomized to one of the two groups. Randomization will be performed centrally by the trial coordinators using an online randomization module (Ennov Clinical®) in a 1:1 allocation ratio between IVF and conversion to IUI, as shown in Fig. 1. Moreover, randomization will be dynamic, and stratified by: center, number of mature follicles (2, 3 or 4), and age (< 40 or ≥ 40 years). | |
| Allocation concealment mechanism | 16b | CF 16a | |
| Implementation | 16c | The patient who wishes to be enrolled will sign the consent form, and will be randomized to one of the two groups. Randomization will be performed centrally by the trial coordinators using an online randomization module (Ennov Clinical®) in a 1:1 allocation ratio between IVF and conversion to IUI, as shown in Fig. 1. Moreover, randomization will be dynamic, and stratified by: center, number of mature follicles (2, 3 or 4), and age (< 40 or ≥ 40 years). | |
| Blinding (masking) | 17a | Not applicable | |
|  | 17b | Not applicable | |
| **Methods: Data collection, management, and analysis** | | | |
| Data collection methods | 18a | All data from the trial will be compiled in an Electronic Case Report Form (eCRF). Only people involved in the trial will have access to the data, via a username and a password. No patient identifying information will be stored, and only the first letter of the surname and of the last name will be kept, without any mention of the full name or date of birth. Patient code will be composed of the participating center’s number and another number assigned by that center. Each participating center will store its own data for the duration set by regulation for this type of studies, under the center’s investigator’s responsibility. Only investigators from Angers University Hospital (AUH) will have access to patient data from other centers on the eCRF. Therefore, for centers that cannot complete patient follow-up at 12 months, patients’ coordinates will be retrieved from the eCRF by AUH investigators and personnel in charge of patient follow-up. At the end of the trial, all data will be deleted. Approval from the CCTIRS (Comité consultatif sur le traitement de l'information en matière de recherche) (Advisory committee on treatment of information in research in the field of healthcare) and the CNIL (Commission nationale informatique et liberté) (National Commission on Informatics and Liberty) have been requested. | |
|  | 18b | Not applicable | |
| Data management | 19 | All data from the trial will be compiled in an Electronic Case Report Form (eCRF). Only people involved in the trial will have access to the data, via a username and a password. No patient identifying information will be stored, and only the first letter of the surname and of the last name will be kept, without any mention of the full name or date of birth. Patient code will be composed of the participating center’s number and another number assigned by that center. Each participating center will store its own data for the duration set by regulation for this type of studies, under the center’s investigator’s responsibility. Only investigators from Angers University Hospital (AUH) will have access to patient data from other centers on the eCRF. Therefore, for centers that cannot complete patient follow-up at 12 months, patients’ coordinates will be retrieved from the eCRF by AUH investigators and personnel in charge of patient follow-up. At the end of the trial, all data will be deleted. Approval from the CCTIRS (Comité consultatif sur le traitement de l'information en matière de recherche) (Advisory committee on treatment of information in research in the field of healthcare) and the CNIL (Commission nationale informatique et liberté) (National Commission on Informatics and Liberty) have been requested. | |
| Statistical methods | 20a | | We will perform a descriptive analysis of the population’s characteristics. Categorical variables will be expressed as numbers with percentages, and compared using Pearson’s chi-square test or Fisher’s exact test. Continuous variables will be reported as mean values and standard deviations, or medians with 25th and 75th percentile, and compared using Student’s t-test or Mann-Whitney’s non parametric test. All statistical tests will be bilateral and a p-value <0.05 will be considered statistically significant. We will perform an intention-to-treat and a per-protocol analysis of all outcomes. For the primary outcome, conversion to IUI will be considered non-inferior to IVF if the upper bound of the one-sided 95% confidence interval of the difference in live birth rates between the two arms (the rate in IVF minus the rate in conversion to IUI) is ≤5%. The primary outcome will be tested using the raw difference in live birth rates, and no complex model incorporating the stratification variables will be used. |
|  | 20b | The dynamic randomization on these variables will ensure the variables are well-balanced. Subgroup analysis will be performed according to patients’ age (<40 vs ≥40 years), the number of mature follicles (2 vs 3 vs 4), and patients considered poor ovarian responders according to the Bologna criteria. On the other hand, the cost-effectiveness analysis will be performed on an intention to treat basis (the hypothesis being that conversion to IUI is superior to IVF). | |
|  | 20c | Data related to patients lost to follow-up or prematurely excluded from the study due to non-adherence will be analysed, even in cases where the consent was later withdrawn by the patient, unless the patient opposes the use of the information. | |
| **Methods: Monitoring** | | | |
| Data monitoring | 21a | | Given the nature of the study and the absence of undesirable side effects related to the interventions, we did not assemble a data monitoring committee (DMC). |
|  | 21b | | No interim analysis is planned. |
| Harms | 22 | | We do not expect to have undesirable or serious undesirable events related to the study and its proceedings. Indeed, all procedures included in this study (COS and its monitoring, ovulation trigger, oocyte retrieval, embryo transfer, IUI) are standard procedures and will be performed in accordance with the national guidelines and recommendations. The only part that is specific to our RCT and that is not considered part of routine practice is the telephone call that will be made to patients 12 months after enrollment in order to collect information on the evolution of pregnancy. This might be a sensitive topic, especially for couples who did not have a healthy live birth, because of pregnancy loss or complications of pregnancy. The personnel in charge of this part will be trained in how to approach the couples and how to deal with the special situations. Moreover, we will have a psychologist available for couples to consult when needed.  All adverse events related to the oocyte retrieval (bleeding, hemoperitoneum, infection, peritonitis, pelvic abscess…) and the anesthesia during the retrieval will be recorded. We will also document whether the complications required any additional treatment, procedure or hospitalization, and add them to the treatment cost. |
| Auditing | 23 | | Trial conduct will be audited in each of the participating centres, once a year, independently from investigators and the sponsor. We will verify that the investigator thoroughly explains the study to the participating subjects, that patients are handed the information letter, and that they sign the consent form before participating. |
| Ethics and dissemination | | | |
| Research ethics approval | 24 | The ethics committee of Lyon South-East II (registration number: 2017-23-2) has granted ethics approval for this study. In the event of additional studies performing sub-analyses based on our database, all the investigators should keep the results confidential until these are publicly available, and they cannot publish any data related to the database without the approval of the principle investigator. During the inclusion visit, the investigator will thoroughly explain the study and hand the patient an information letter, written in an easily accessible language. The patient who wishes to be enrolled will sign the consent form, and will be randomized to one of the two groups. | |
| Protocol amendments | 25 | In cases of protocol modifications (eg. changes to eligibility criteria, outcomes, analyses), a request will be first sent to the ethics committee (REC). If approved, the information will be sent to all investigators. | |
| Consent or assent | 26a | The investigator or the co-investigators of the study | |
|  | 26b | Not applicable | |
| Confidentiality | 27 | All data from the trial will be compiled in an Electronic Case Report Form (eCRF). Only people involved in the trial will have access to the data, via a username and a password. No patient identifying information will be stored, and only the first letter of the surname and of the last name will be kept, without any mention of the full name or date of birth. Patient code will be composed of the participating center’s number and another number assigned by that center. Each participating center will store its own data for the duration set by regulation for this type of studies, under the center’s investigator’s responsibility. Only investigators from Angers University Hospital (AUH) will have access to patient data from other centers on the eCRF. Therefore, for centers that cannot complete patient follow-up at 12 months, patients’ coordinates will be retrieved from the eCRF by AUH investigators and personnel in charge of patient follow-up. At the end of the trial, all data will be deleted. Approval from the CCTIRS (Comité consultatif sur le traitement de l'information en matière de recherche) (Advisory committee on treatment of information in research in the field of healthcare) and the CNIL (Commission nationale informatique et liberté) (National Commission on Informatics and Liberty) have been requested. | |
| Declaration of interests | 28 | All authors are nothing to disclose | |
| Access to data | 29 | Only investigators from Angers University Hospital (AUH) will have access to patient data from other centers on the eCRF. | |
| Ancillary and post-trial care | 30 | Not applicable | |
| Dissemination policy | 31a | All trial results, oral communications and publications will be managed by the investigator coordinator in collaboration with the main co-investigators and participating scientists. | |
|  | 31b | All investigators and participating clinicians will be eligible for co-authorship, depending on their contribution to the study, as well as the methodologist, the biostatistician, and the involved researchers. | |
|  | 31c | Not applicable | |
| Appendices |  |  | |
| Informed consent materials | 32 | CF Model consent form of the study in additional file 2. | |
| Biological specimens | 33 | Not applicable | |

*It is strongly recommended that this checklist be read in conjunction with the SPIRIT 2013 Explanation & Elaboration for important clarification on the items. Amendments to the protocol should be tracked and dated. The SPIRIT checklist is copyrighted by the SPIRIT Group under the Creative Commons “[Attribution-NonCommercial-NoDerivs 3.0 Unported](http://www.creativecommons.org/licenses/by-nc-nd/3.0/)” license.
